# A method for obtaining flexible broccoli varieties for sustainable agriculture

**DOI:** 10.1186/s12863-020-00846-2

**Published:** 2020-05-07

**Authors:** Simona Ciancaleoni, Valeria Negri

**Affiliations:** Dipartimento di Scienze Agrarie, Alimentari e Ambientali (DSA3), Università degli Studi di Perugia, Borgo XX Giugno 74, 06121 Perugia, Italy

**Keywords:** *Brassica oleracea* L. var. *italica*, Evolutionary breeding program, Populations, Sustainable agriculture, Genetic diversity, Adaptation

## Abstract

**Background:**

The use of high inputs in agriculture resulted in few varieties (hybrids and pure lines) used in all agricultural systems. Also varieties of vegetables, including broccoli, for organic and low-input agriculture, are almost exclusively hybrids, since there are very few specific breeding programs and varieties for sustainable agriculture systems.

A strategy to overcome this issue is the adoption of specific breeding programs for developing heterogeneous varieties (i.e. synthetics, open pollinated varieties, composite cross populations and mixtures). In fact, heterogeneous varieties are able to evolve and adapt to specific agro-climatic conditions.

The aim of this study was to develop a method (an Evolutionary Breeding Program, EBP) for obtaining heterogeneous varieties of broccoli and test its efficiency in developing highly diverse varieties, as needed in sustainable agriculture.

A synthetic variety originated from a landrace was multiplied in different environments for 3 cycles and morpho-phenological and genetic diversity of the derived populations were assessed.

**Results:**

The presented results are the first and unique indication about the efficiency of a short-time EBP for an allogamous species like broccoli.

Few morphological changes were observed among varieties multiplied in different environments with different agro-climatic conditions. This could be probably due to the initial genetic diversity of the landrace from which the populations were selected and also to the great plasticity of the crop. However, SSR data highlighted a genetic differentiation among populations multiplied for two/three years across Europe and in Central Italy, that was not so evident when considering morphological data only.

**Conclusions:**

Few years of multiplication in different environments resulted in genetically differentiated broccoli populations that nonetheless preserved the original genetic diversity and productivity level and appear to evolve in relationship to different environments: the applied EBP is useful for developing heterogeneous materials for sustainable agriculture.

## Background

*Brassica oleracea* L. (2n = 2x = 18) is an economically important vegetable and its world annual commercial production (cauliflower and broccoli) is over 25 Mt, currently cultivated on about 1.4 Mha worldwide [1]. China is the first country in producing broccoli and cauliflower (10 million tons), meanwhile, in Italy, the estimated production of both vegetables is about 390,000 tons [1] of which 10% is obtained in organic agriculture [2]. Among the different *B. oleracea* subspecies, broccoli (*B. oleracea* L. var. *italica* Plenck, also identified as *B. oleracea* L. spp*. capitata* L. (DC.) convar*. Botrytis* (L.) Alef*.* var*. italica* Plenck [3]) is an important source of vitamins, minerals and antioxidants.

Currently, organic farmers largely depend on broccoli varieties bred for high external input in conventional farming systems [4, 5], and in particular, broccoli varieties are almost exclusively hybrids [5], due to lack of specific breeding programs and varieties for sustainable (organic and low-input) agriculture.

Suitable varieties for sustainable agriculture should have specific characteristics (as yield stability under different agro-climatic conditions, resistances to biotic and abiotic stresses, competition ability with weeds) in order to avoid the utilisation of external input like pesticides, herbicides and chemical fertilizers [5, 6]. In the context of sustainable agriculture, to allow varieties to evolve and adapt to specific agro-climatic conditions is also important [7]. This is possible only when heterogeneous varieties (i.e. landraces, synthetics, open pollinated varieties, composite cross populations, mixtures) are used and reproduced on-farm for generations.

As a consequence, breeding programs specifically adopted for sustainable agriculture should consider all the above. Among many breeding approaches, the Evolutionary plant Breeding Program (EBP), combining natural and artificial selection in target environments, is suggested to be effective in obtaining heterogeneous varieties for organic and low-input agriculture [8–13].

The success of an EBP, like that of any other breeding program, is mainly due the genetic diversity of the initial materials. For example, EBP in cereals has been based on composite cross populations (CCP) or mixtures [8, 10, 13, 14] that can be originated from old varieties or landraces. Also for allogamous species, genetically variable varieties, like open pollinated, synthetic varieties or landraces, which have advantages in terms of yield stability and resistance [15], have been recommended. In particular, landraces could be the best starting materials for developing varieties suitable for organic and low-input agriculture [5, 16] due their great genetic diversity that provides a buffer against environment fluctuations and adaptation to specific environments across time.

An assessment of genetic diversity is therefore essential for the organization, development and control of a breeding program. The genetic diversity and structure of populations under selection can be efficiently evaluated by molecular markers. Depending on their type (i.e. neutral or related-to-gene markers) they can also provide information about the intensity of both natural and human selection and about the genetic divergence of populations developed across years and environments. To this purpose, different types of molecular markers have been used. For example, genetic changes in maize composite populations under Reciprocal Recurrent Selection (RRS) were analyzed by using Restriction Fragment Length Polymorphism (RFLP) [17, 18], Simple Sequence Repeats (SSR) [19–21] and Single Nucleotide Polymorphism (SNP) [22]. SSR were also used to evaluate the genetic changes in different maize populations obtained after about 20 years of stratified mass selection on two historical Portuguese populations [23]. Considering autogamous species, the genetic evolution over time and space of wheat CCP was assessed by RFLP [24, 25] and by SSR [26] and that of a barley CCP by using SSR [13].

Agronomic performances of different broccoli heterogeneous varieties or other broccoli breeding materials for sustainable agriculture have been already reported [5, 27–29], but few data are available on genetic diversity of broccoli breeding materials [30–32] and on their evolution and adaptations under different agro-climatic conditions.

Considering this lack of knowledge, the aims of this paper were to i) describe a EBP method for developing flexible broccoli varieties (i.e. populations, for sustainable agriculture), ii) assess the adaptation potential and the morpho-phenological and genetic changes of the populations developed with this method in different environments and iii) in the adaptation area across years.

## Results

### Comparisons of populations multiplied for up to two cycles in different environments

#### Morpho-phenological characterisation and differentiation

The ANOVA analysis showed that the entries differ for all of the morpho-phenological traits recorded with great differences generally observed between the populations and three of the hybrids (HH, HHH, HHHH) (Table 1). For the traits weight of the main head (*YLD*) and number of secondary heads (*HN*) the populations derived from the initial synthetic by multiplication for two years were not different from each other. In particular, *YLD* values ranged from 11.05 g (Syn2-TER) to 324.58 g (HHH), as populations formed a little main head and many (up to 36) secondary heads while most of the hybrids did not form any secondary heads HHH, among the hybrids, and Syn2-FR, among the populations, showed the highest total yield (324.8 and 266.2 g, respectively).

**Table 1:** Average values and standard deviations of morpho-phenological trait for the entries (two years of multiplication)

	*DH*	*DM*	*HN*	*YLD*	*M-YLD*	*T-YLD*	*HeH*	*HeW*	*SW*	*PH*	*PD*	*V*
LR	203a	215b	34.00a	26.73c	6.47bcd	242.04bc	8.50a	3.50cd	1.26bcd	50.78a	66.78b	6.47ab
SD	±4.83	±2.21	±10.95	±11.91	±1.93	±84.08	±1.56	±1.06	±0.37	±10.14	±14.58	±1.50
Syn1-PG	204a	215b	33.00a	28.39c	6.10cd	228.65cd	8.71a	3.72cd	1.29bcd	50.63a	62.17bc	6.48ab
SD	±4.63	±3.31	±8.23	±11.43	±1.69	±71.16	±1.71	±1.36	±0.39	±7.27	±6.30	±1.25
Syn2-PG	191c	207d	33.00a	38.97c	5.36d	213.74cd	9.11a	4.35cd	1.33bcd	44.83ab	80.39a	6.83ab
SD	±2.29	±0.21	±8.57	±34.79	±1.80	±70.67	±3.07	±2.39	±0.44	±9.73	±10.46	1.59±
Syn2-GR	197b	219a	36.00a	22.09c	3.32e	144.49de	5.97b	5.34c	1.36bcd	48.63a	63.38bc	7.04a
SD	±0.00	±2.72	±8.45	±11.30	±0.89	±59.16	±1.81	±1.06	±1.23	±6.76	±11.69	±1.49
Syn2-TER	188c	207d	35.00a	11.05c	1.93e	81.05e	6.41b	5.15cd	0.94d	39.35b	48.90d	4.00c
SD	±2.55	±1.79	±11.13	±4.55	±0.67	±37.27	±1.13	±1.18	±0.20	±10.23	±12.84	±1.52
Syn2-FR	204a	216b	29.00a	44.46c	7.97a	266.72abc	8.81a	3.89cd	1.53b	48.97a	66.48b	6.55ab
SD	±4.94	±1.90	±9.15	±91.06	±2.96	±139.01	±3.01	±2.94	±0.53	±8.55	±11.67	±1.52
Syn2-UK	203a	215b	31.00a	23.41c	6.87abc	233.56c	8.70a	3.29d	1.08cd	45.63ab	64.38bc	5.72b
SD	±450	±1.85	±10.61	±8.67	±1.88	±83.01	±1.54	±1.06	±0.30	±9.86	±8.17	±1.40
H	192c	212b	29.00a	30.42c	7.57ab	252.04abc	9.59a	5.04cd	1.46bc	44.81ab	36.31e	2.94c
SD	±0.00	±3.18	±9.50	±9.07	±2.26	±119.79	±2.14	±1.24	±0.24	±9.53	±9.54	±0.51
HH	101e	134f	0.00b	312.62a	0.00f	312.62ab	9.58a	14.53a	3.45a	29.28c	57.69cd	–
SD	±1.83	±3.55	±0.00	±104.09	±0.00	±104.09	±2.17	±3.11	±0.44	±5.31	±7.12	–
HHH	107d	137e	0.00b	324.58a	0.00f	324.58a	9.64a	14.15a	3.61a	28.60cd	64.52bc	–
SD	±8.64	±5.32	±0.00	±120.88	±0.00	±120.88	±2.41	±4.10	±0.39	±4.20	±7.15	–
HHHH	107d	140e	0.00b	224.68b	0.00f	229.82cd	7.82ab	11.37b	3.69a	22.05d	71.33ab	–
SD	±5.54	±6.77	±0.00	±105.55	±0.00	±105.41	±1.70	±2.86	±0.81	±5.83	±11.76	–

*B. oleracea* var. *italica* original LR, Syn1-PG, its derived populations by two years of multiplication across Europe and hybrid controls

Averages followed by the same letters are not significantly different at *P* < 0.05 (Tukey HSD)

Considering the inflorescence emission and maturation date (*DH* range = 101–204 dd; *DM* range = 134–219 dd), generally, the latest were the populations, the earliest the hybrids, with the exception of H. LR, Syn1-PG, Syn2-FR, Syn2-UK and Syn2-GR were the latest for inflorescence emission date, with Syn2-GR also late in maturation. Regarding traits related to head characteristics, generally, hybrids showed higher values than populations: *HeH* range = 6.41–9.59 cm, (Syn2-TER and H, respectively), *HeW* range = 3.29–14.53 cm (Syn2-UK and HH, respectively) and *SW* range = 0.94–3.69 cm (Syn2-TER and HHHH, respectively). The hybrids showed the lowest plant height, while LR and Syn1-PG the highest (*PH* range = 22.05–50.78 cm, HHHH and LR, respectively). Regarding plant diameter (*PD* range = 36.31–80.39 cm, in H and Syn2-PG, respectively), all the entries showed similar values, with the exception of H, HH and Syn2-TER showing the lowest values. No differences were found among populations regarding vigour with the only exception of Syn2-TER (4.0) which was the less vigorous one.

**Fig. 1:**
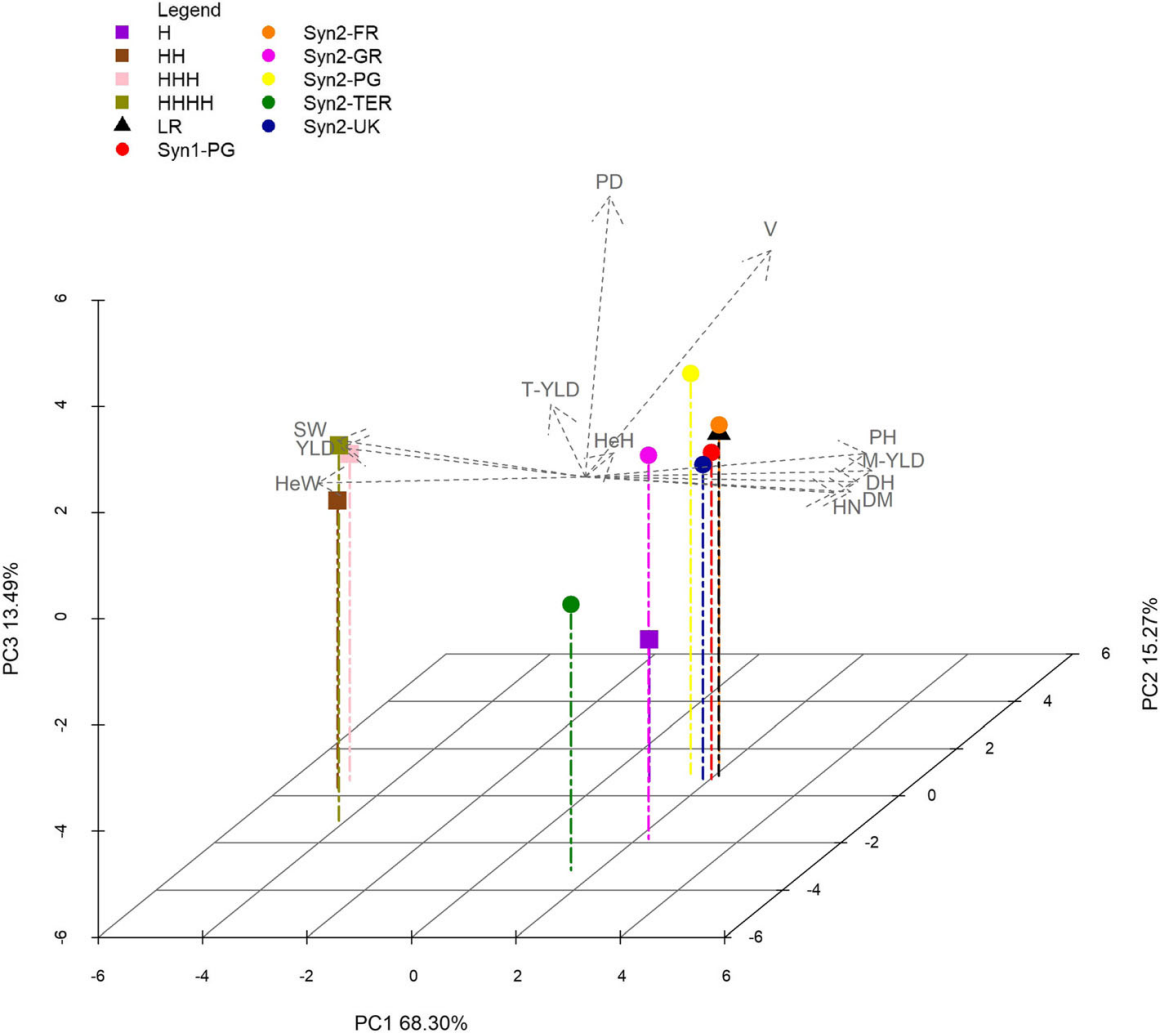
PCA based on morpho-phenological traits recorded for each entry (two years of multiplications). *B. oleracea* var. *italica* original LR, Syn1-PG, its derived populations by two years of multiplication across Europe and hybrid controls. Coloured symbols represent the different entries analysed as from the legend, grey dotted arrow represent the morpho-phenological traits coded as from Table 4

The first two PCA axes accounted for 83.57% of total data variability, with the addition of the third axis the 97.06% of the total variability was explained (Fig. 1). The entries were clustered in two groups: the first one included HH, HHH and HHHH, while the second included the populations with Syn2-TER and H as subgroups of this cluster. Most of the morpho-phenological traits had large effects on the ordination of entries (as indicated by the lengths of their vectors along the first two Axis).

The variables were clustered into four groups: the first one included *YLD*, *SW* and *HeW*, the second one included *T-YLD* and *HeH*, the third one *PD* and *V*, while the last group included all the other variables. Within each group, variables were highly positively correlated, but the variables of the first group were negatively correlated to those belonging to the third group. The variables of the second group were not correlated with those included in the third group, while they were positively correlated with those belonging to the first group (Fig. 1).

#### Genetic characterisation and differentiation

Averages and standard errors relative to the main genetic parameters calculated by entry over the 22 SSR loci are reported in Table 2.

**Table 2:** Averages and standard errors relative to the main genetic parameters calculated by entry (two years of multiplication)

Entry		***N***	***Na***	***Ne***	***Ho***	***He***	***F***
LR	Mean	30.91d	3.50a	2.37a	0.48a	0.51a	0.04a
	SE	0.262	0.327	0.212	0.044	0.039	0.054
Syn1-PG	Mean	31.32cd	3.50a	2.38a	0.46ab	0.52a	0.09a
	SE	0.179	0.307	0.188	0.041	0.037	0.055
Syn2-PG	Mean	31.36bcd	2.95ab	2.18ab	0.53a	0.49a	-0.05a
	SE	0.124	0.223	0.154	0.045	0.034	0.068
Syn2-GR	Mean	31.86abc	2.68b	1.93ab	0.39abc	0.43a	0.05a
	SE	0.100	0.202	0.143	0.037	0.037	0.052
Syn2-TER	Mean	31.77abc	2.45bc	1.86bc	0.43abc	0.42a	-0.03a
	SE	0.113	0.205	0.088	0.048	0.041	0.052
Syn2-UK	Mean	31.95ab	2.59b	2.03ab	0.46ab	0.45a	-0.03a
	SE	0.045	0.204	0.138	0.048	0.042	0.057
Syn2-FR	Mean	31.00d	3.00b	1.78bcd	0.42abc	0.38ab	0.07a
	SE	0.208	0.255	0.126	0.057	0.041	0.079
H	Mean	32.00a	1.50d	1.34de	0.22bc	0.18c	-0.17ab
	SE	0.000	0.127	0.094	0.068	0.048	0.113
HH	Mean	32.00a	1.41d	1.41cde	0.41abc	0.21c	−1.00c
	SE	0.000	0.107	0.107	0.107	0.052	0.000
HHH	Mean	32.00a	1.27d	1.19e	0.19c	0.10c	−0.51b
	SE	0.000	0.097	0.084	0.084	0.042	0.177
HHHH	Mean	31.91abc	1.82cd	1.43cde	0.34abc	0.24bc	−0.21ab
	SE	0.063	0.156	0.090	0.077	0.047	0.127

Mean and standard errors relative to the number of successfully analysed genotypes (*N*), observed (*Na*), and effective alleles (*Ne*), observed heterozygosity (*Ho*), expected heterozygosity (*He*) and Fixation Index (*F*) worked out for original LR, Syn1-PG, its derived populations by two years of multiplication across Europe and hybrid controls on the basis of data recorded overall for the 22 markers used

All the entries were characterised by similar number of alleles (*N*). In general, *Na* was higher than *Ne* in all the entries. In populations, *Na* ranged from 2.45 (±0.205, Syn2-TER) to 3.50 (±0.327, LR) and *Ne* ranged from 1.78 (±0.126, Syn2-FR) to 2.38 (±0.188, Syn1-PG), hybrids generally had lower values of *Na* and *Ne* than the populations.

Populations did not differ for *Ho* and *He*, while obviously differed from hybrids.

Fixation index (*F*) values were close to 0 for all the populations. The existence of negative values in some populations and in all the hybrids hinted to a heterozygosity excess.

Finally, all *Fst* values obtained from pairwise comparisons were highly significant (*P* < 0.01), with the only exception of the comparison between LR and Syn1-PG (Table 3). Considering two years of multiplication, significant pairwise *Fst* comparisons between the LR and derived populations increased from 0.026 to 0.066. In addition, a crescendo of differentiation was found between Syn1-PG and populations derived by multiplication in other environments (from 0.039 between Syn1-PG and Syn2-GR, to 0.070 between Syn1-PG and Syn2-TER).

**Table 3:** *Fst* values calculated for each pairwise comparison between original LR, Syn1-PG, its derived populations by two and three years of multiplication across Europe and in Central Italy, respectively, and hybrid controls

	LR	Syn1-PG	Syn2-PG	Syn3-PG	Syn2-GR	Syn3-GR	Syn2-TER	Syn3-TER	Syn2-UK	Syn2-FR	H	HH	HHH	HHHH
LR	–	n.s.												
Syn1-PG	0.000	–												
Syn2-PG	0.022	0.022	–											
Syn3-PG	0.041	0.044	0.029	–										
Syn2-GR	0.042	0.039	0.046	0.066	–									
Syn3-GR	0.093	0.086	0.131	0.132	0.107	–								
Syn2-TER	0.066	0.070	0.103	0.124	0.096	0.104	–							
Syn3-TER	0.100	0.099	0.108	0.120	0.124	0.115	0.159	–						
Syn2-UK	0.057	0.050	0.073	–	0.076	–	0.104	–	–					
Syn2-FR	0.056	0.064	0.082	–	0.101	–	0.108	–	0.116	–				
H	0.247	0.258	0.268	0.317	0.276	0.314	0.339	0.313	0.348	0.281	–			
HH	0.221	0.221	0.239	0.282	0.246	0.305	0.308	0.285	0.278	0.268	0.461	–		
HHH	0.281	0.288	0.299	0.329	0.350	0.390	0.388	0.356	0.360	0.317	0.543	0.259	–	
HHHH	0.213	0.215	0.224	0.251	0.265	0.304	0.295	0.288	0.278	0.262	0.425	0.175	0.162	–

All comparison are significant *P* < 0.002, only LR vs Syn1-PG *Fst* is not significant *P* > 0.05

*n.s* not significant

Similarly, a crescendo of differentiation was found between Syn2-PG and the same generation of multiplication in different environments (from 0.046 between Syn2-PG and Syn2-GR to 0.103 between Syn2-PG and Syn2-TER). The highest differentiation value was found between Syn2-FR and Syn2-TER, populations which were multiplied in very contrasting environments. As expected, the genetic differentiation between hybrids and between populations and hybrids was very high.

AMOVA (not shown) worked out on the total data showed that among- and within- population diversity accounted for 33 and 67% of total genetic variation, respectively. However, when it was worked out only considering the LR and its derived populations, the proportions were 11 and 82%, respectively.

NJ tree results showed the presence of two main clusters (Additional file 4: Figure S3). A cluster included most of the individuals belonging to populations while the other one to the hybrids. The first cluster showed others, although not completely differentiated, sub-clusters: Syn2-TER, Syn2-FR and Syn2-UK genotypes mostly clustered together. The within-population genetic variation was evident in populations, while hybrids generally showed a high genetic uniformity in accordance with their genetic structure.

**Fig. 2:**
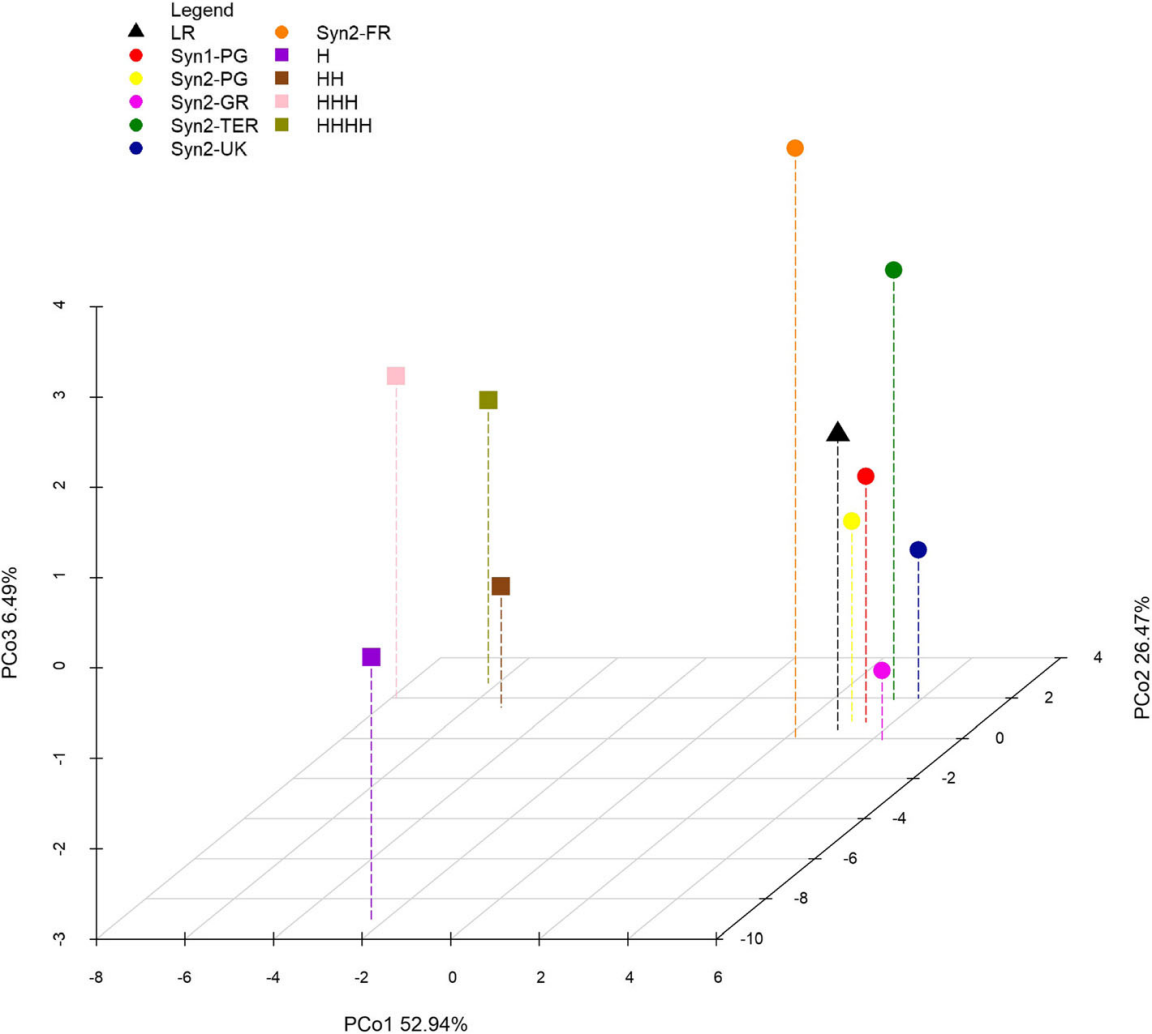
PCoA based on average genetic distance of entries (two years of multiplication). *B. oleracea* var. *italica* original LR, Syn1-PG, its derived populations by two years of multiplication across Europe and hybrid controls. Coloured symbols represent the different entries analysed as from the legend

The first three components of the PCoA accounted for 85.90% of genetic variation (Fig. 2). PCo1 (52.94%) clearly separated populations from hybrids, while PCo2 (26.47%) separated the hybrid H from the other entries. PCo3 (6.49%) mainly discriminated Syn2-FR and Syn2-TER from Syn2-UK and Syn2-GR.

### Comparison of populations multiplied for up to three cycles in Central Italy

#### Morpho-phenological and genetic characterisation

The ANOVA analysis showed that the assessed entries differ for many of the morpho-phenological traits recorded (Additional file 5: Table S2). All populations were clearly discriminated from HH, HHH and HHHH. However, populations multiplied at the same site, with very few exceptions, did not show much differences and, generally, all populations obtained after three cycles of multiplication showed similar values to those found in the populations obtained after two cycles of multiplication. Worth of note is only that Syn2-GR was different from Syn3-GR and Syn2-TER from Syn3-TER for *HeH*, Syn2-PG was different from the LR and the other populations multiplied in Perugia for *DH, DM* and *PD* and, finally, Syn3-PG was different from LR, Syn1-PG and Syn2-PG for *V*.

The PCA (with the first three axes accounting for 96.54% of total data variation) and the biplot showed that the three hybrids and hybrid H with populations clustered separately and that the variable clustering and relationship were similar to what previous observed (i.e. considering the morpho-phenological comparisons of populations multiplied for up two cycles in different environments, Additional file 6: Figure S4).

Averages and standard errors relative to the main genetic parameters calculated by entry over the 22 SSR loci are reported in Additional file 7: Table S3. A part differences between entries, populations obtained with three years of multiplication did not significantly differ from those obtained with two years of multiplication for all genetic parameters, with the exception of *Na* observed in Syn2-GR and Syn3-GR. Finally, pairwise *Fst* comparisons (Table 3) ranged from 0.005 to 0.159 between populations. Population differentiation increased across locations and generations of multiplication. The highest differentiation was found between Syn2-TER and Syn3-TER.

NJ tree results, beside a clear differentiation between populations and hybrids, showed that Syn2-TER, Syn3-TER, Syn3-GR, and partially Syn3-PG appeared to belong to four different and distinguished clusters (Additional file 8: Figure S5). The first three components of the PCoA accounted for 82.63% of genetic variation (Additional file 9: Figure S6). Populations and H were clearly discriminated from HH, HHH and HHHH. Finally, Syn3-GR, Syn2-TER and Syn3-TER were clearly differentiated from all the other populations.

## Discussion

To the best of our knowledge this is the first insight into the morphological and genetic short-term evolution and local adaptation of broccoli populations developed by a EBP for sustainable agriculture.

The populations here studied could be compared to natural populations, that are mainly represented by genotypes derived from the most fit plants and that mainly evolve in relationship to environmental pressures, because the EBP applied (i.e. the choice of the most vigorous plants for reproduction) favoured plants with the largest heads and then the greatest production of seed in each environment.

Although several experiences of evolutionary breeding for autogamous species have been reported by different authors ([8, 12] and references therein, [33, 34]), a few EBP have been registered for allogamous species like broccoli [23, 35, 36].

As for the comparison of populations developed in different countries, the selection pressures (those applied by different researchers by choosing three plants during multiplication of populations and those linked to different pedo-climatic conditions) did not seem to have heavily changed the morpho-phenological traits and the performances of populations in comparison with LR from which they derived. The populations showed few morphological differences probably due to their intrinsic plasticity, the initial genetic diversity of LR and the mating system of the species, factors that, combined, maintain a large variability [30]. In addition, it is possible that the most vigorous chosen plants per generation had the highest heterozygosity and different alleles were consequently maintained across generations. Similar results were also found by [35, 36] who evaluated the morphological changes in spinach and maize in few years of multiplication in different environments, respectively.

Only populations multiplied in TER were morphologically well distinguished from the other populations. TER is an environment that showed minimum temperatures lower than the others among December and March: these environmental factors could have negatively influenced the vegetative stage of plants and consequently the reproductive stage, since from December to March the heads appear and then proceed to maturation.

Also, the EBP here described (selection and mating of the best tree plants for two years) did not seem to have resulted in any inbreeding depression, suggesting that the genetic diversity remained high.

In spite of that, genetic data showed a differentiation among populations that morphological data did not assess. Only two years of multiplication caused a low to moderate differentiation. Worth of note is the progressive increase in differentiation values observed passing from different population generations and from a multiplication environment to another.

Of course, a longer breeding period or a different selection method than the one implemented in this study could have allowed a greater differentiation among populations, as it happens in the process of broccoli LR differentiation [31]. For example, as suggested by results of [23], in which the final versions of two maize populations showed low differentiation from the original populations, mass selection resulted less selective than a RRS implemented for a similar period [18]. In addition, in both maize mass and RSS, a long breeding program increased the genetic differentiation among original and final breeding populations [18, 37], probably as a consequence of allele fixation [18, 38]. In fact, if 2–4 cycles of RRS slightly reduce diversity, with an allele loss up to 11% [20, 22, 39], 11–12 cycles of RRS reduce diversity much more, with an allele loss up to 37% [17, 40].

Our genetic results were similar to those found by [23] (comparing two open-pollinated maize populations undergone to stratified mass selection for 19 and 25 years) and to those found by [20] who (comparing two maize synthetic varieties after two cycles RRS) found that the genetic differentiation among versions of the same population was very low.

In this study, the short selection period and/or the low pressure breeding method resulted in the maintenance of a high intra-population genetic diversity.

Also in maize a study of molecular changes during intra- and inter- recurrent selection on one adapted and one non adapted populations suggested that natural selection acted stronger than breeding in differentiating the populations obtained in the non adapted population only [21]. Similarly, passing from a multiplication environment to another, we observed an increase of differentiation between the same population generations. Although in this study on broccoli environmental pressures did not strongly affected genetic diversity as in maize [21], indeed they played a role in differentiating populations.

The comparisons of populations developed in Central Italy for up to three cycles confirmed what observed comparing populations developed in different countries. The SSR data highlighted a genetic differentiation among populations that was not evident with only morphological data. With respect to populations obtained after two cycles of multiplication, one more year of multiplication determined a greater genetic differentiation among populations: genetic distance between Syn1-PG and Syn3-PG was greater than between Syn1-PG and Syn2-PG. Also, the highest *Fst* values were those between the final versions of populations multiplied in different environments.

In addition, genetic data showed that the environment of adaptation (Perugia, the environment in which LR is grown and the original selection was carried out in order to develop the Syn1-PG) was less selective than the others. In fact, even if the genetic distance among the Syn1-PG and the derived populations was increasing along years, the increase was larger in the TER and GR environments, which are not the adaptation environments.

During an EBP the selective pressure of environments is crucial, but time is also an important factor in driving the oscillations of the allele frequencies and the subsequent adaptation of the genotypes.

## Conclusions

One of the aims of this study was to assess the adaptation potential of a heterogeneous variety when multiplied in different environments. Although a longer multiplication period would be necessary to have a final confirmation, the obtained data show that a high genetic diversity is maintained all over the tested multiplication environments and in spite of their pedological and climatic differences in at least three years. This diversity can then continue to evolve in response to different selection pressures in each environment in the future, as it has always happened with any introduction of variable materials in different environments in the past.

Few years of multiplication in different environments resulted in differentiated populations which, nonetheless, preserved the original genetic diversity and productivity level and appear able to evolve in relationship to different environments.

As such the EBP here described also appears to be a good method to develop broccoli materials (populations) for sustainable agricultural systems, those systems where the use of external input (pesticides, irrigation, fertilisers that help a crop facing adversities) is limited or absent.

In sustainable agricultural systems, protection from biotic and abiotic stress (which are variable from year to year and from a situation to another) should rely on the diverse response of the single different genotypes at a certain moment and, consequently, on the ability of the crop, as a whole, to change its composition across time, making the crop community diverse and resistant/tolerant across time and situation. This changing ability is also important because sustainable systems often have very specific characteristics in term of soil fertility, type of occurring biotic and abiotic stress, agronomic and protection techniques applied by farmers (also in relation to their own skills and preferences). In most cases none is similar to another. Finally, it is also important because we are living in a period of rapid and unpredictable climatic changes, with the connected changes of biological forms negatively affecting crops.

For all these reasons, in sustainable agriculture populations like those developed in this study, which are able to evolve across time and locations, appear to be much more suitable materials than hybrids and greater efforts than presently carried out should be applied to develop and diffuse them. Of course, where external inputs can counteract biotic and abiotic constrains and the cultivation environment is uniform, hybrid varieties can be the most useful materials.

## Materials and methods

### Plant breeding procedure

Aiming to develop materials suitable for organic agriculture (i.e. possessing the adaptation and the intrinsic diversity needed to suit a particular environment) we specifically focused our work on a landrace (LR) instead of on other materials.

We chose a sprouting broccoli LR from Umbria (Central Italy) that is named “Zolfino”, highly appreciated on the local market and generally grown under low-input conditions. The LR is characterised by late flowering and maturation: requiring low winter temperatures for flower induction, it is harvested in a period spanning from beginning of March to mid-April. Initially the main head, then the lateral buds are harvested in the period.

The initial material of LR was obtained by a farmer family which cultivated it since generations. After the farmer abandoned his own population, this Department (DSA3) continued its cultivation by yearly reproducing the seed and applying the selection procedures of the mother plants (MP) suggested by the farmer: the most vigorous 2 or 3 MP, among the 25 usually grown, are chosen and intercrossed, while the field is cleared from other *B. oleracea* plants, since all the species varieties are interfertile. It should be noted that this procedure is common across Europe for farmers reproducing their *B. oleracea* LR (V. Negri personal communication).

**Fig. 3:**
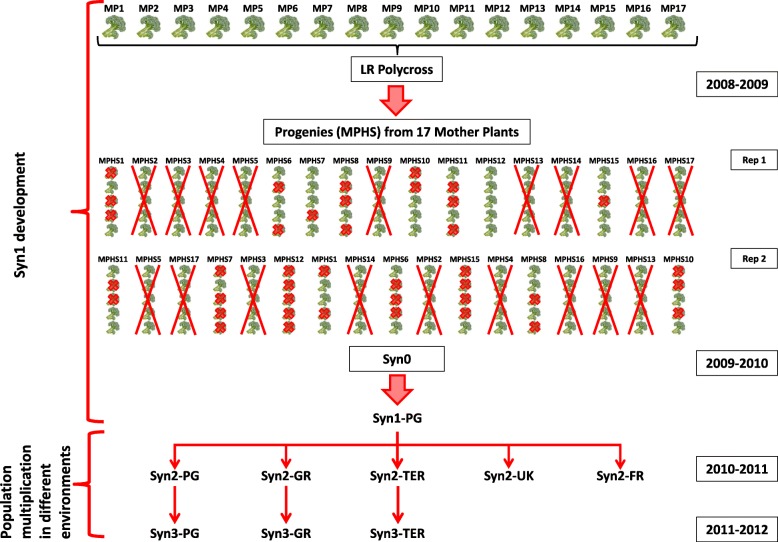
Schematic representation of the EBP from Syn1-PG development to multiplications of populations

Starting a breeding program to develop an initial synthetic in 2009, we asked the farmer to choose 17 MP among the 25 grown instead of the usual number (Fig. 3). The following year, 10 plants for each of the 17 MP half sib progenies (MPHS) were grown in a trial (5 plants for each MPHS in each of 2 replicates, for a total of 170 plants). Such a high number of plants was adopted in order to maintain the initial LR diversity as much as possible. Among them, the farmer was asked to select, according to his personal opinion, the best 8 MPHS and then, within each of them, the best 5 plants. Accordingly, a synthetic variety (Syn0) made of 40 plants from the initial 8 MPHS, that mirrors the original LR, was obtained.

The multiplication of Syn0 produced a Syn1-PG (2009–2010), the initial (foundation) material of this study.

To start the EBP the Syn1-PG seed was then multiplied in isolation in different areas of Central Italy, in France and in United Kingdom as follows (Fig. 3):

i) for two consecutive years at DSA3 (Perugia, Umbria Region) in the same area of its origin giving rise to two populations named Syn2-PG (2010–2011) and Syn3-PG (2011–2012), respectively;
ii) for two consecutive years in two other areas of Central Italy with different pedo-climatic conditions from Perugia: Grosseto (Tuscany) and Terminillo (Lazio), giving the populations named Syn2-GR, Syn3-GR and Syn2-TER and Syn3-TER, respectively;
iii) for one year only (2010–2011) in United Kingdom giving a population named Syn2-UK;
iv) for one year only (2010–2011) in France giving a population named Syn2-FR.

Each multiplication environment (GR, TER, UK and FR) is different from PG for several pedo-climatic traits (Additional file 1: Figure S1, [27]). Such different environmental conditions were purposely chosen to assess the degree of restriction in genetic diversity due to selective pressure.

As it is usual for this type of LR, in all sites the seeds obtained from each multiplication cycle was sowed in pots at the beginning of July and the plantlets were transplanted in the experimental field at the beginning of September. The seed was harvested in June in Italy, and about a month later in France and United Kingdom.

In each multiplication cycle and in each environment only the three most vigorous plants (in agreement with farmer indications) were intercrossed in order to obtain seed. The inflorescences of the other plants were cut before flowering in order to evaluate agronomic traits of the commercial heads.

Each year, the obtained seed was sent to DSA3 in order to store it in its Genebank (FAO code ITA363) for the successive analyses (see below).

For each environment of multiplication and for the environment of final agronomic trial, climatic data, i.e. average daily temperature, average of maximum daily temperatures, average of minimum daily temperatures (°C) and total rainfall precipitations (mm) (obtained from near-by weather stations), were recorded from sowing to harvest (Additional file 1: Figure S1).

### Evaluated plant material

The materials obtained from multiplication in different environments was used to assess the occurrence morpho-phenological and genetic changes.

Firstly, particularly to understand the effects of multiplication in different countries, we assessed the diversity of genotypes belonging to 11 entries of broccoli: seven populations and four hybrids used as controls. Specifically: the Umbrian LR, the Syn1-PG, its five two year multiplications, Syn2-PG, Syn2-GR, Syn2-TER, Syn2-UK and Syn2-FR, and the hybrids Santee (Elsomseed), Ironman (Monsanto), Belstar (Bejo Seeds) and Natura Vallata (Cooperativa Agricola Cesenate), named H, HH, HHH and HHHH, respectively.

Secondly, we assessed other three populations obtained in Central Italy with a further multiplication cycle (Syn3-PG, Syn3-TER and Syn3-GR) in order to obtain information about further evolutionary processes eventually occurred.

### Assessment of morpho-phenological changes occurred with multiplications

In order to assess eventual morpho-phenological changes, all the obtained multiplications were evaluated in the same environment.

Seeds from the 14 broccoli entries were sown in pots at the beginning of July and the obtained plantlets were transplanted in a DSA3 experimental field in Perugia (Umbria, Italy) at the beginning of September. A total of 448 individual plants were evaluated. Plants were arranged by using a randomised block design with four replications. In each plot, eight plants of each entry were grown, for a total of 32 plants evaluated per entry. Spacing among plants was set at 1 × 1 m because the plants are very vigorous and this spacing is generally used by farmers growing the “Zolfino” LR (Additional file 2: Figure S2). The management practices applied to the agronomical trial were similar to those used in organic agriculture conditions in the area. Fertilization was ensured by pre-implant livestock manuring, by using 1 t ha^− 1^ of the commercial product NUTEX LETAME (Agroqualità, 3% N and 3% P_2_O_5_ content) that was incorporated by using a rotary cultivator. As above, this is the fertilization level usually applied by farmers. Weed control was always performed by hand-weeding and no chemical control of pathogens and insects was applied. Broccoli were irrigated immediately after transplanting and during the following month, aiming at fully restoring crop evapotranspiration.

Twelve morpho-phenological traits were recorded for each plant on all entries (Table 4).

**Table 4:** Morphological and phenological traits, their description and codes

Trait	Description of method of detection	Code
Days to heading	Number of days from sowing to head emission (dd)	*DH*
Days to maturity	Number of days from sowing to maturity of the main head (dd)	*DM*
Head number	Number of secondary heads at maturity of the main head (n)	*HN*
Yield	Fresh weight of the main head (g)	*YLD*
Mean Yield	Average weight of heads (*T-YLD/(HN + 1)*)	*M-YLD*
Total-Yield	Fresh weight of the total heads per plant (g)	*T-YLD*
Head height	Longitudinal dimension of the main head (cm)	*HeH*
Head width	Width of the main head (mm)	*HeW*
Stem width	Width of the main head stem (mm)	*SW*
Plant height	Height of the plant (cm)	*PH*
Plant diameter	Diameter of the plant (cm)	*PD*
Vigour	Plant vigour (Score from min = 0 to max = 9)	*V*

To determine the significance of the sources of variation, the recorded data were processed by analysis of variance (ANOVA) using a linear model, where an individual trait value T_ij_ of the levels i of the effect “Genotype” (G), and j of the effect “Block” (B), is: T_ij_ = m + G_i_ + B_j_ + e_ij_, where m is the grand mean and e_ij_ is the experimental error, and means were separated using the Tukey Honest Significant Difference (HSD), as implemented in the HSD.test() function of the “agricolae” package in R [41, 42].

In order to study the correlation pattern of the considered morpho-phenological traits and the differences among entries, a principal components analysis (PCA) was performed by using the function PCA() of the “FactoMineR” package [43]. PCA is a factor analytic technique that is used for the ordination of observations in a reduced rank space. It is used to visualise the weight of variables in determining entry ordination (as shown by vector lengths, by angles between vectors and by the reciprocal positioning of variable vectors and entry symbols). As a preliminary step before performing a PCA on the complete dataset, the missing values were imputed with the Principal Components Analysis model by the function imputePCA() of the “missMDA” package [44].

### Assessment of genetic changes occurred with multiplications

#### Genomic DNA extraction

Fifty mg of fresh leaf tissue were collected from each of the 448 plants (32 plants for each of 14 entries) and high quality DNA was isolated using the DNeasy 96 Plant Kit (Quiagen). DNA quality and concentration were checked by spectrophotometry using NanoDrop 2000 (Thermo Scientific) and 1.0% (w/v) agarose gel separation.

#### Microsatellite analysis

Broccoli individuals were genotyped using 17 putative neutral microsatellite markers (SSR) [45–47] and five microsatellites related to genes involved in flowering control and cold stress response (EST-SSR) [48] (Additional file 3: Table S1). According to their position on *B. oleracea* chromosomes, the selected markers are not in linkage (i.e. for each chromosome, the two selected markers are located on the two different arms). All the DNA amplifications were carried out as described in [49]. Obtained amplicons were separated in a 3130xl capillary sequencer (Applied Biosystems), sized according to the internal size standard GeneScanTM23 500LIZ® (Applied Biosystems), visualised and scored using the GENEMAPPER software (Applied Biosystems).

For each SSR marker the number of alleles and allele size range were recorded. The number of successfully analysed (*N*) and observed (*Na*) genotypes, the effective (*Ne*) number of observed alleles, observed (*Ho*), as well as the expected (*He*) heterozygosity and the Fixation Index (*F*), were worked out by entry (i.e. LR, Syn1-PG, its derived populations and hybrid controls) with the use of GENALEX software [50]. For each parameter, the average values were compared by using the Tukey Honest Significant Difference (HSD) as implemented in the HSD.test() function of the “agricolae” package in R [41, 42].

In order to assess the components of molecular variation, an Analysis of Molecular Variance (AMOVA) was worked out following the method of [51] as implemented in GENALEX software [50].

Pairwise comparison *Fst* values and two Genetic Distance (GD) matrices between individuals following the formula of [52] were calculated using GENALEX software [50]. Two GD-based Neighbour Joining (NJ) trees were drawn using the on-line software iTOL [53]. To better understand the genetic relationship among entries, two Principal Coordinate Analysis (PCoA) were also worked out by using the same distance matrices and drawn by “scatterplot3d” package in R [42, 54].

## Supplementary information

**Additional file 1: Figure S1.** Monthly precipitation (mm) and mean of minimum, maximum and average temperature (°C). Data registered during the multiplication seasons in Perugia in 2009/2010 (PG 2009/2010), in Central Italy (Perugia, Grosseto e Terminillo) in 2010/2011 and 2011/2012 (PG 2010/2011, PG 2011/2012, GR 2010/2011, GR 2011/2012, TER 2010/2011 and TER 2011/2012), in United Kingdom in 2010/2011 (UK 2010/2011) and in France in 2010/2011 (FR 2010/2011), and during the final agronomic trial carried out in Perugia (PG 2013/2014). The bar chart refers to monthly precipitation, lines with squares, diamonds and circles refer to maximum, average and minimum temperature, respectively.

**Additional file 2: Figure S2.** Final agronomic trial carried out in the DSA3 experimental field in Perugia (Umbria, Italy). Broccoli multiplications carried out across Europe and in Central Italy were grown in the DSA3 experimental field in Perugia. The populations tested were: LR, Syn1-PG, Syn2-PG, Syn3-PG, Syn2-GR, Syn3-GR, Syn2-TER, Syn3-TER, Syn2-UK, Syn2-FR. H, HH, HHH and HHHH are the hybrid varieties used as controls.

**Additional file 3: Table S1.** SSR markers used for analyze genetic diversity of broccoli original LR, Syn1-PG, its derived populations and hybrid controls. Bibliographic reference codes, genebank entry, primer sequences, repeated motif, linkage group (LG), and band range (in base pairs) relative to the 22 microsatellites used (^*1*^Love et al. 2004; ^*2*^Li et al. 2011; ^*3*^Cheng at al. 2009; ^*4*^Aksoy et al. 2013).

**Additional file 4: Figure S3.** Neighbour-joining tree of genetic distances of the entries (two years of multiplication). *B. oleracea* var. *italica* original LR, Syn1-PG, its derived population by two years of multiplication across Europe and hybrid controls are coded with abbreviations and coloured with different colours as follows: LR-black, Syn1-PG-red, Syn2-PG-yellow, Syn2-GR-purple, Syn2-TER-dark green, Syn2-UK-blue, Syn2-FR-orange, H-violet, HH-brown, HHH-pink, HHHH-saddlebrown. Each individual of each entry are code whit a different number.

**Additional file 5: Table S2.** Average values and standard deviations of morpho-phenological traits for the entries (three years of multiplication). *B. oleracea* var. *italica* original LR, Syn1-PG, its derived populations by three years of multiplication in Central Italy and hybrid controls. Averages followed by the same letters are not significantly different at *P* < 0.05 (Tukey HSD).

**Additional file 6: Figure S4.** PCA based on morpho-phenological traits recorded for each entries (three years of multiplication). *B. oleracea* var. *italica* original LR, Syn1-PG, its derived populations by three years of multiplication in Central Italy and hybrid controls. Coloured symbols represent the different entries analysed as from the legend, grey dotted arrow represent the morpho-phenological traits coded as from Table 4.

**Additional file 7: Table S3.** Averages and standard errors relative to the main genetic parameters calculated by entry (three years of multiplication). Number of successfully analysed genotypes (*N*), observed (*Na*), and effective alleles (*Ne*), observed heterozygosity (*Ho*), expected heterozygosity (*He*), and Fixation Index (*F*) worked out for original LR, Syn1-PG, its derived populations by three years of multiplication in Central Italy and hybrid controls on the basis of data recorded overall for the 22 markers used.

**Additional file 8: Figure S5.** Neighbour-joining tree of genetic distances of the entries (three years of multiplication). *B. oleracea* var. *italica* original LR, Syn1-PG, its derived populations by three years of multiplication in Central Italy and hybrid controls are coded with abbreviations and coloured with different colours as follows: LR-black, Syn1-PG-red, Syn2-PG-yellow, Syn3-PG-light green, Syn2-GR-purple, Syn3-GR-grey, Syn2-TER-dark green, Syn3-TER-light blue, H-violet, HH-brown, HHH-pink, HHHH-saddlebrown. Each individual of each entry are code whit a different number.

**Additional file 9: Figure S6.** PCoA based on average genetic distance of the entries (three years of multiplication). *B. oleracea* var. *italica* original LR, Syn1-PG, its derived populations by three years of multiplication in Central Italy and hybrid controls. Coloured symbols represent the different entries analysed as from the legend.

## Data Availability

The datasets used and/or analyzed during the current study are available from the corresponding author on reasonable request.

## Abbreviations

DSA: Dipartimento di Scienze Agrarie, Alimentari e Ambientali
EBP: Evolutionary Breeding Program
CCP: Composite Cross Populations
RRS: Reciprocal Recurrent Selection
SSR: Simple Sequence Repeats
RFLP: Restriction Fragment Length Polymorphism
SNP: Single Nucleotide Polymorphism
LR: Landrace
MP: Mother Plant
MPHS: Mother Plant Half Sib
Syn: synthetic variety
PG: Perugia Environment
GR: Grosseto Environment
TER: Terminillo Environment
UK: United Kingdom Environment
FR: France Environment
H: Hybrid variety
HH: Hybrid variety
HHH: Hybrid variety
HHHH: Hybrid variety
DH: Days to heading
DM: Days to maturity
HN: Head number
YLD: Yield
M-YLD: Mean Yield
T-YLD: Total-Yield
HeH: Head height
HeW: Head width
SW: Stem width
PH: Plant height
PD: Plant diameter
V: Vigour
PCA: Principal components analysis
PCoA: Principal Coordinate Analysis
N: Number of successfully analysed genotypes
Na: Number of successfully genotypes
Ne: Effective number of observed alleles
Ho: Observed heterozygosity
He: Expected heterozygosity
F: Fixation Index
GD: Genetic distance
NJ: Neighbour Joining
