# Supplementary figures and images for: A method for obtaining flexible broccoli varieties for sustainable agriculture
Source: BMC Genet. 2020 May 7;21:51. doi: 10.1186/s12863-020-00846-2 (PMC7203864; doi:10.1186/s12863-020-00846-2)

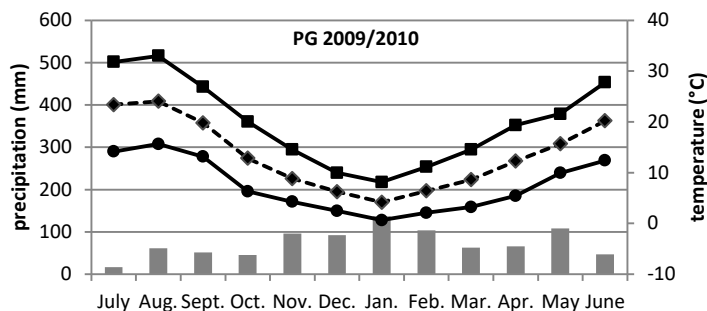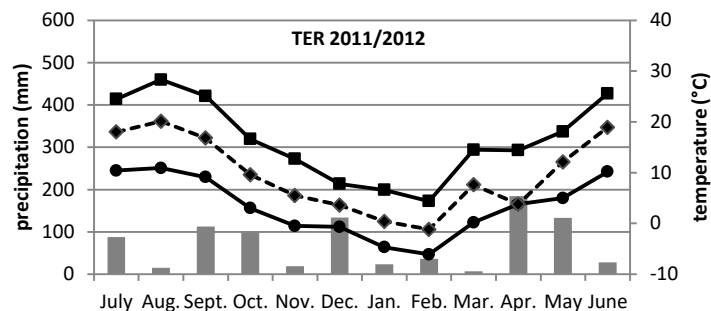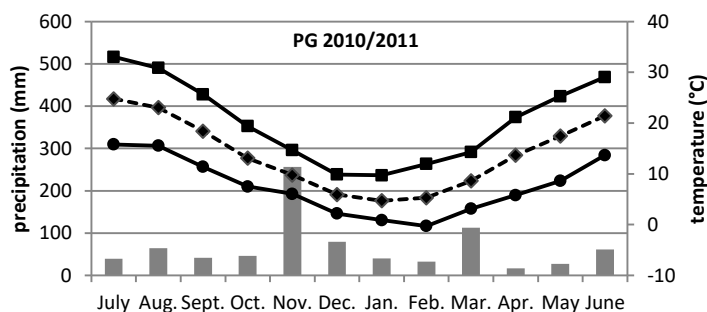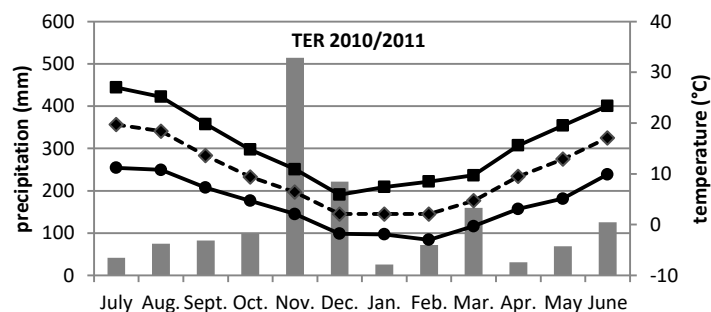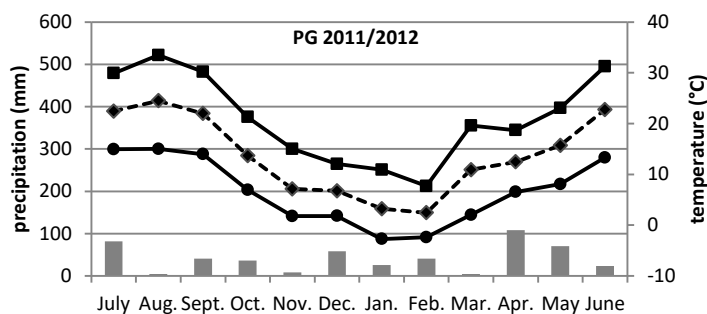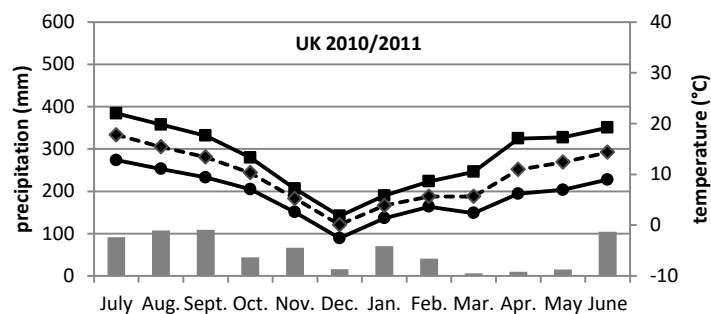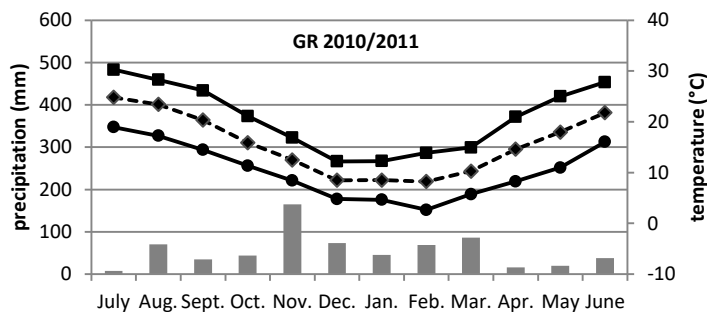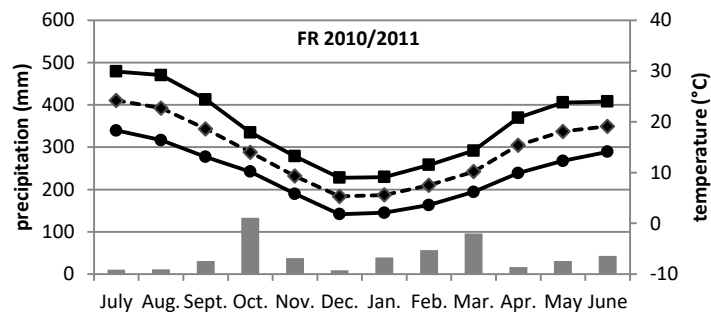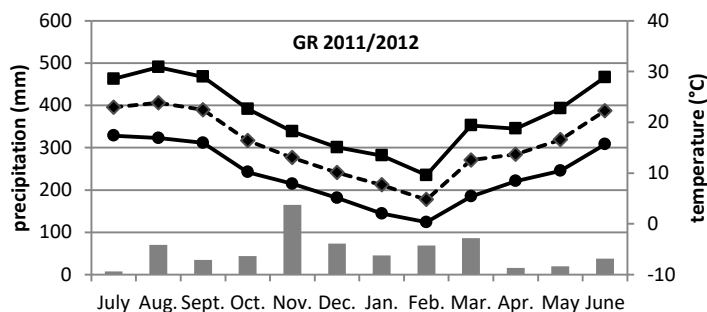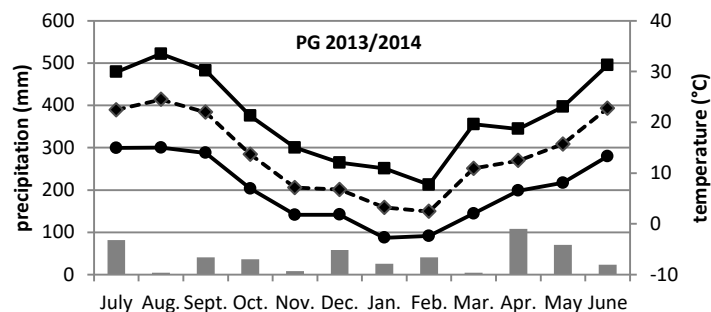

Supplement: Supplementary file 1 — Additional file 1: Figure S1. Monthly precipitation (mm) and mean of minimum, maximum and average temperature (°C). Data registered during the multiplication seasons in Perugia in 2009/2010 (PG 2009/2010), in Central Italy (Perugia, Grosseto e Terminillo) in 2010/2011 and 2011/2012 (PG 2010/2011, PG 2011/2012, GR 2010/2011, GR 2011/2012, TER 2010/2011 and TER 2011/2012), in United Kingdom in 2010/2011 (UK 2010/2011) and in France in 2010/2011 (FR 2010/2011), and during the final agronomic trial carried out in Perugia (PG 2013/2014). The bar chart refers to monthly precipitation, lines with squares, diamonds and circles refer to maximum, average and minimum temperature, respectively. [file 12863_2020_846_MOESM1_ESM.pdf]

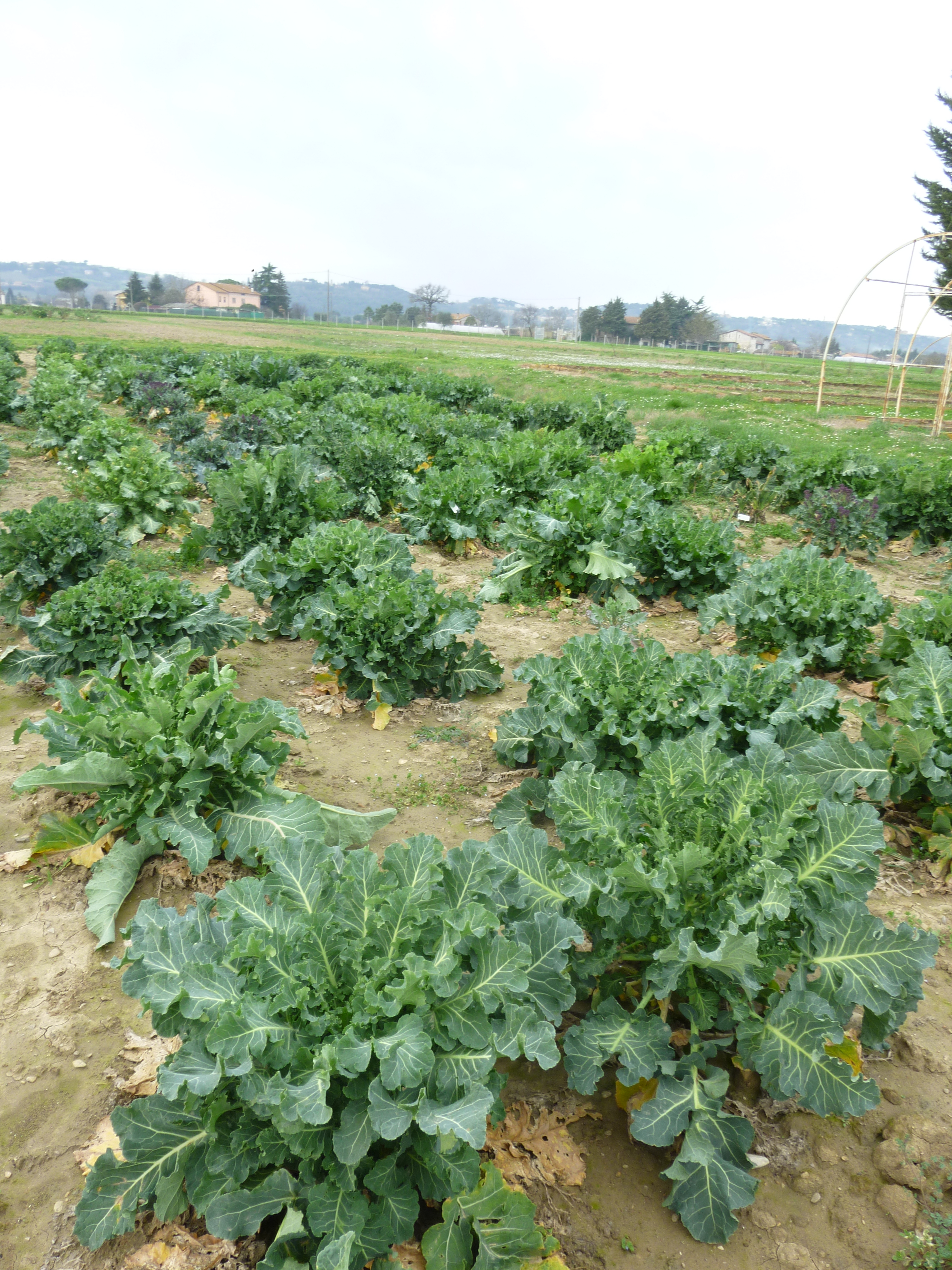

Supplement: Supplementary file 2 — Additional file 2: Figure S2. Final agronomic trial carried out in the DSA3 experimental field in Perugia (Umbria, Italy). Broccoli multiplications carried out across Europe and in Central Italy were grown in the DSA3 experimental field in Perugia. The populations tested were: LR, Syn1-PG, Syn2-PG, Syn3-PG, Syn2-GR, Syn3-GR, Syn2-TER, Syn3-TER, Syn2-UK, Syn2-FR. H, HH, HHH and HHHH are the hybrid varieties used as controls. [file 12863_2020_846_MOESM2_ESM.tiff]

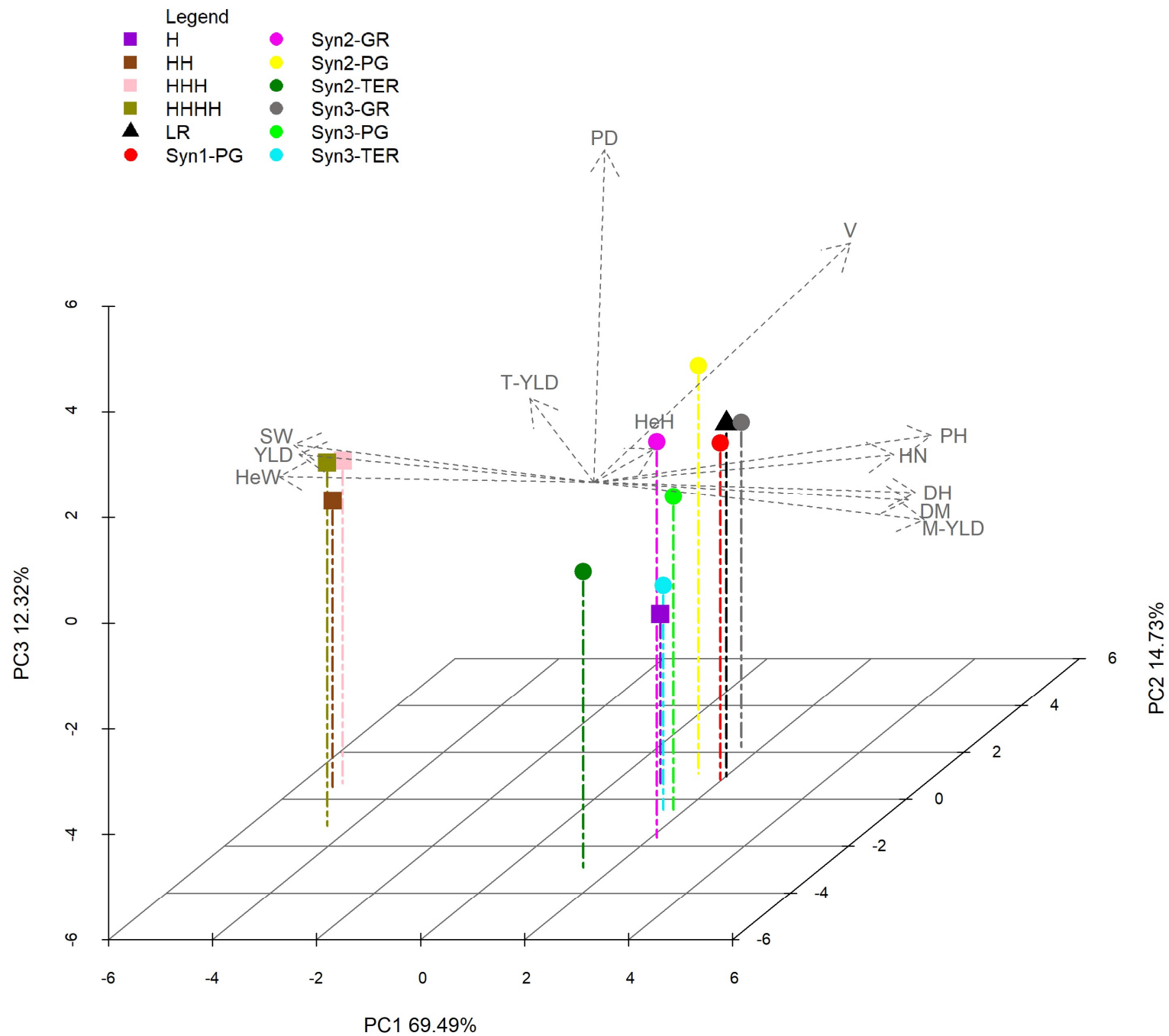

Supplement: Supplementary file 6 — Additional file 6: Figure S4. PCA based on morpho-phenological traits recorded for each entries (three years of multiplication). B. oleracea var. italica original LR, Syn1-PG, its derived populations by three years of multiplication in Central Italy and hybrid controls. Coloured symbols represent the different entries analysed as from the legend, grey dotted arrow represent the morpho-phenological traits coded as from Table 4. [file 12863_2020_846_MOESM6_ESM.pdf]

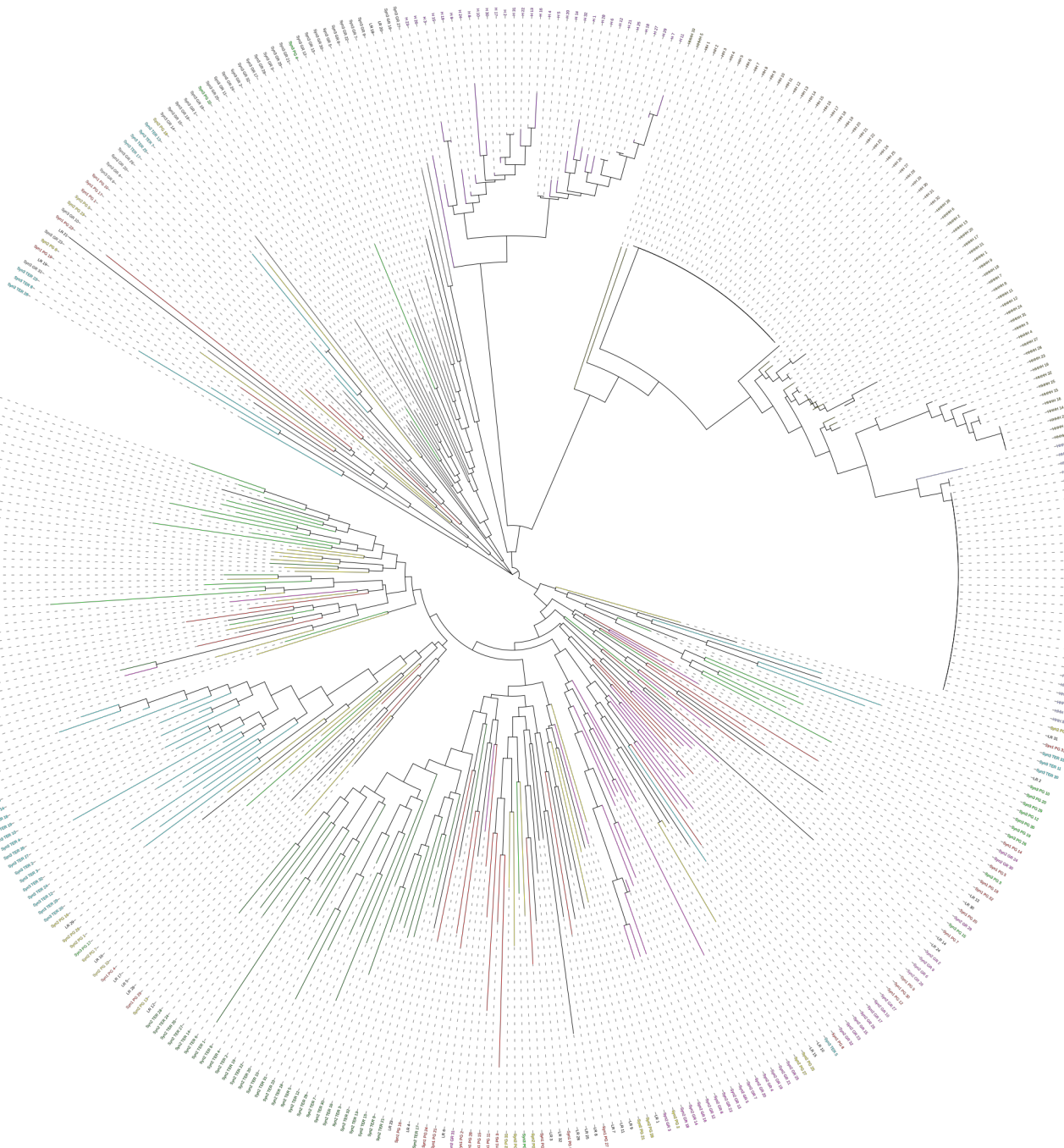

Supplement: Supplementary file 8 — Additional file 8: Figure S5. Neighbour-joining tree of genetic distances of the entries (three years of multiplication). B. oleracea var. italica original LR, Syn1-PG, its derived populations by three years of multiplication in Central Italy and hybrid controls are coded with abbreviations and coloured with different colours as follows: LR-black, Syn1-PG-red, Syn2-PG-yellow, Syn3-PG-light green, Syn2-GR-purple, Syn3-GR-grey, Syn2-TER-dark green, Syn3-TER-light blue, H-violet, HH-brown, HHH-pink, HHHH-saddlebrown. Each individual of each entry are code whit a different number. [file 12863_2020_846_MOESM8_ESM.pdf]

- Legend
- LR
  - Syn1-PG
  - Syn2-PG
  - Syn3-PG
  - Syn2-GR
  - Syn3-GR
  - Syn2-TER
  - Syn3-TER
  - H
  - HH
  - HHH
  - HHHH

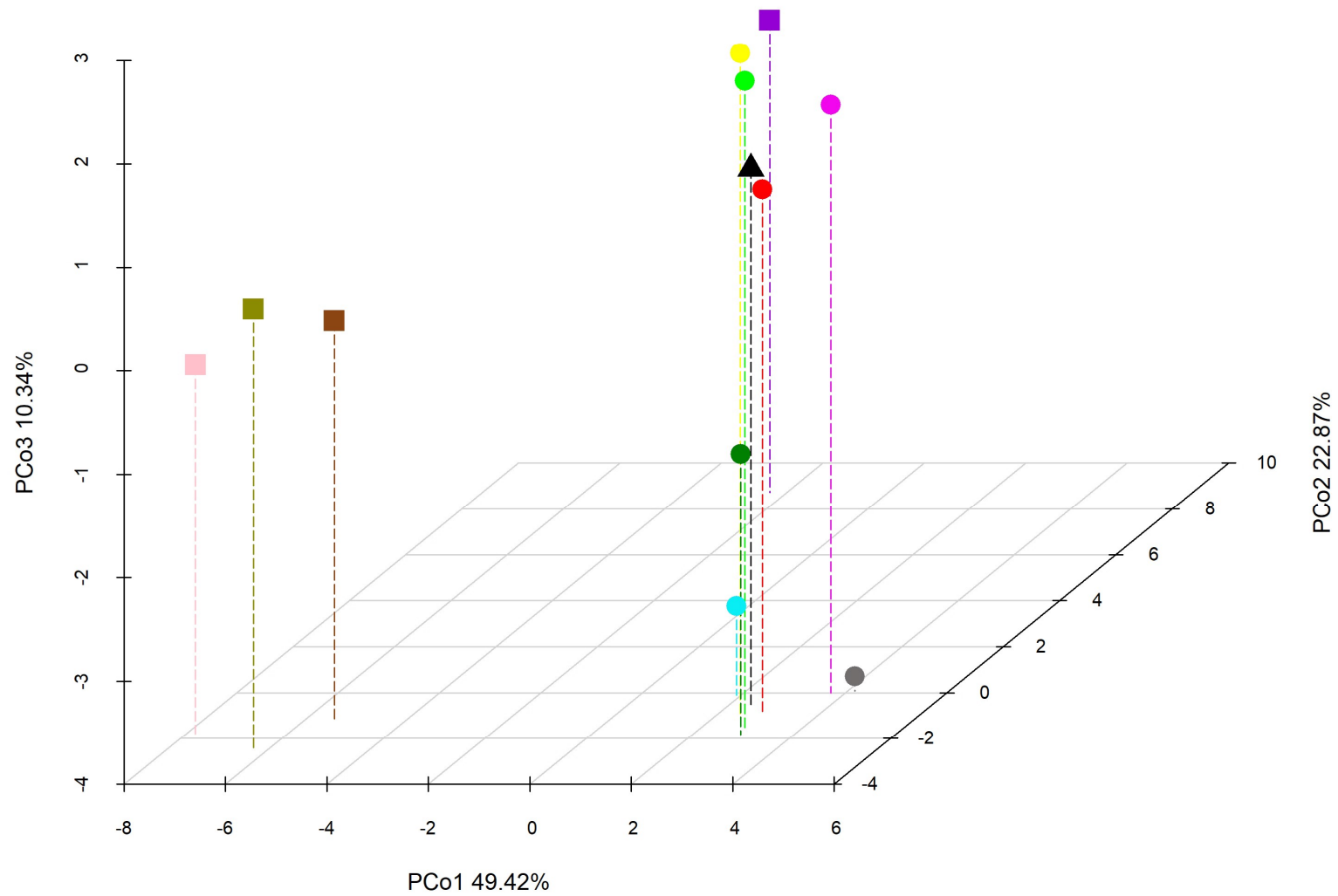

Supplement: Supplementary file 9 — Additional file 9: Figure S6. PCoA based on average genetic distance of the entries (three years of multiplication). B. oleracea var. italica original LR, Syn1-PG, its derived populations by three years of multiplication in Central Italy and hybrid controls. Coloured symbols represent the different entries analysed as from the legend. [file 12863_2020_846_MOESM9_ESM.pdf]
