# Supplementary material for: A method for obtaining flexible broccoli varieties for sustainable agriculture
Source: BMC Genet. 2020 May 7;21:51. doi: 10.1186/s12863-020-00846-2 (PMC7203864; doi:10.1186/s12863-020-00846-2)
Supplement: Supplementary file 3 — Additional file 3: Table S1. SSR markers used for analyze genetic diversity of broccoli original LR, Syn1-PG, its derived populations and hybrid controls. Bibliographic reference codes, genebank entry, primer sequences, repeated motif, linkage group (LG), and band range (in base pairs) relative to the 22 microsatellites used (1Love et al. 2004; 2Li et al. 2011; 3Cheng at al. 2009; 4Aksoy et al. 2013). [file 12863_2020_846_MOESM3_ESM.pdf]

SSR	Entry	Primers sequence (5'-3')	Repeat Motif	LG	Band range
SSR6[1]	Ol12G04b	F: CGAACATCTTAGGCCGAATC<br>R: GGTTAACCTGCGGGATATTG	(TC)29	8	100-161
SSR9[1]	Ol10B01	F: CCTCTTCAGTCGAGGTCTGG<br>R: AATTTGGAAACAGAGTCGCC	(GA)20	7	162-169
SSR10[1]	Na12C08	F: GCAAACGATTTGTTTACCCG<br>R: CGTGTAGGGTGATCTAGATGGG	(CT)50	1	268-354
SSR 20[1]	Ol12F02a	F: GGCCCATTGATATGGAGATG<br>R: CATTTCTCAATGATGAATAGT	(TC)29	5	127-255
SSR22[1]	Ol10B01	F: GCCTTAGATTAGATGGTCGCC<br>R: ACTTCAGCTCCGATTTGCC	(CT)29	6	139-143
SSR23	Na12A02b	F: AGCCTTGTTGCTTTTCAACG<br>R: AGTGAATCGATGATCTCGCC	(CT)16	6	163-167
SSR25[1]	Ol10H04	F: TCACCCCTCTATATCCACCC<br>R:CAGAATCTGCCTGAACATCG	(CT)29	7	158-217
SSR31[2]	BoGMS1042	F: ATAGTGAATAATGGAAGGCTG<br>R: GAGAGAGGAGAGAACAGAGGA	(TC)14	1	182-194
SSR37[3]	BoGMS0726	F: GTTCCGAGGGTTGTTCTT<br>R: CCATCAGGTTCAGCCATAC	(AT)18	2	203-209
SSR39[2]	BoGMS0702	F: CGTAATGGTGAAGATACTCGG<br>R: TCTAATCAAGAGCGTGTGGT	(AT)18	3	262-270
SSR42[2]	BoGMS1465	F: GAGGTGTTGGATACTGTGCT<br>R: TGTTGTTGGTGTTAGTGGTG	(GAA)8	3	284-299
SSR45[2]	BoGMS0560	F: ATGAAGAAGTGTTTGGTGAG<br>R: AAGATTGATGGAAGCAAGAA	(GAA)14	4	279-303
SSR47[3]	BnGMS681	F: GTCGAAGATTGTTGTCAGGT<br>R: TTCACGAAGAACCCTAGAAA	(GT)8	4	142-144
SSR49[2]	BoGMS0949	F: CTCCTCCTCCTTTCATCTTC<br>R: TTCGTCTCCCTTCTCTGTAA	(TC)15	5	164-204
SSR54[2]	BoGMS0354	F: CTGACAATGACCAAGATAAGG<br>R: CACGCAACAACAACAACTAC	(TGT)6(GTT)4	8	100-109
SSR58[2]	BoGMS1570	F: TCAAGCCAACGCTACTACA<br>R: TGATGGGTGAACAACTAAACT	(AATA)6	9	192-193
SSR59[2]	BoGMS1467	F: ATGGCTTTGTTCTTCTTTCTT<br>R: GACTTCAGCACGCCTTTC	(CTG)8	9	267-279
B_FLC3-SSR3[4]	AY306125	F: AGGCAAAATCTCTCGTATCTGG<br>R: CTTCTGCTCCACCGATGAAT	(TC)11		206-208
Bo_LFY-SSR7[4]	FJ529019	F: ATTTTCTTTCGCTATGTCTGGC<br>R: TGTGTCGTTTCCATAACTCGAC	(TC)15		182-184
Bo_FT-SSR9[4]	FJ848914	F: GAATCGGTATATTCAAGCCAGTC<br>R: ACCCATGAACACTAAAATCCGT	(TCT)4		220-222
Bo_CRY1-SSR17[4]	AJ344565	F: TTGGGTGAATAATTTCCAAAAG<br>R: TCTTCCTCTGGTGCCCATAC	(T)15(T)13		324-334
Bo_FRI-GN19[4]	JF318402	F: AGCGGTCACAGTTTCTTGTC<br>R: GCATTCCACGCTTGATACTTG	-		380
