# Supplementary material for: A method for obtaining flexible broccoli varieties for sustainable agriculture
Source: BMC Genet. 2020 May 7;21:51. doi: 10.1186/s12863-020-00846-2 (PMC7203864; doi:10.1186/s12863-020-00846-2)
Supplement: Supplementary file 5 — Additional file 5: Table S2. Average values and standard deviations of morpho-phenological traits for the entries (three years of multiplication). B. oleracea var. italica original LR, Syn1-PG, its derived populations by three years of multiplication in Central Italy and hybrid controls. Averages followed by the same letters are not significantly different at P < 0.05 (Tukey HSD). [file 12863_2020_846_MOESM5_ESM.pdf]

	DH	*DM*	*HN*	*YLD*	*M-YLD*	*T-YLD*	*HeH*	*HeW*	*SW*	*PH*	*PD*	*V*
LR	203a	215b	34.00a	26.73c	6.47bcd	242.04bc	8.50a	3.50cd	1.26bcd	50.78a	66.78b	6.47ab
SD	±4.83	±2.21	±10.95	±11.91	±1.93	±84.08	±1.56	±1.06	±0.37	±10.14	±14.58	±1.50
Syn1-PG	204a	215b	33.00a	28.39c	6.10cd	228.65cd	8.71a	3.72cd	1.29bcd	50.63a	62.17bc	6.48ab
SD	±4.63	±3.31	±8.23	±11.43	±1.69	±71.16	±1.71	±1.36	±0.39	±7.27	±6.30	±1.25
Syn2-PG	191c	207d	33.0a	38.97c	5.36d	213.74cd	9.11a	4.35cd	1.33bcd	44.83ab	80.39a	6.83ab
SD	±2.29	±0.21	±8.57	±34.79	±1.80	±70.67	±3.07	±2.39	±0.44	±9.73	±10.46	1.59±
Syn3-PG	204a	215b	29.00ab	19.42c	5.88bc	190.47cde	7.67cd	3.43d	1.01c	43.47abc	59.13cd	5.12bc
SD	±4.58	±5.56	±10.93	±10.13	±1.88	±85.08	±1.90	±0.85	±0.36	±11.03	±12.82	±1.92
Syn2-GR	197b	219a	36.00a	22.09c	3.32e	144.49de	5.97b	5.34c	1.36bcd	48.63a	63.38bc	7.04a
SD	±0.00	±2.72	±8.45	±11.30	±0.89	±59.16	±1.81	±1.06	±1.23	±6.76	±11.69	±1.49
Syn3-GR	201ab	213bc	31.00a	28.08c	7.41ab	260.67abc	10.33a	3.83cd	1.16bc	48.94ab	68.47b	6.00ab
SD	±5.65	±3.49	±10.02	±15.20	±2.15	±101.74	±2.34	±1.07	±0.34	±9.56	±8.23	±1.15
Syn2-TER	188c	207d	35.00a	11.05c	1.93e	81.05e	6.41b	5.15cd	0.94d	39.35b	48.90d	4.00c
SD	±2.55	±1.79	±11.13	±4.55	±0.67	±37.27	±1.13	±1.18	±0.20	±10.23	±12.84	±1.52
Syn3-TER	202a	215b	22.00b	19.88c	7.78a	168.34de	8.43bc	3.34d	1.01c	43.34bc	47.07f	3.379d
SD	±4.12	±1.76	±10.54	±9.74	±4.90	±96.53	±1.98	±1.04	±0.37	±14.39	±12.79	±1.37
H	192c	212b	29.00a	30.42c	7.57ab	252.04abc	9.59a	5.04cd	1.46bc	44.81ab	36.31e	2.94c
SD	±0.00	±3.18	±9.50	±9.07	±2.26	±119.79	±2.14	±1.24	±0.24	±9.53	±9.54	±0.51
HH	101e	134f	0.00b	312.62a	0.00f	312.62ab	9.58a	14.53a	3.45a	29.28c	57.69cd	-
SD	±1.83	±3.55	±0.00	±104.09	±0.00	±104.09	±2.17	±3.11	±0.44	±5.31	±7.12	-
HHH	107d	137e	0.00b	324.58a	0.00f	324.58a	9.64a	14.15a	3.61a	28.60cd	64.52bc	-
SD	±8.64	±5.32	±0.00	±120.88	±0.00	±120.88	±2.41	±4.10	±0.39	±4.20	±7.15	-
HHHH	107d	140e	0.00b	224.68b	0.00f	229.82cd	7.82ab	11.37b	3.69a	22.05d	71.33ab	-
SD	±5.54	±677	±0.00	±105.55	±0.00	±105.41	±1.70	±2.86	±0.81	±5.83	±11.76	-
