# Supplementary material for: A method for obtaining flexible broccoli varieties for sustainable agriculture
Source: BMC Genet. 2020 May 7;21:51. doi: 10.1186/s12863-020-00846-2 (PMC7203864; doi:10.1186/s12863-020-00846-2)
Supplement: Supplementary file 7 — Additional file 7: Table S3. Averages and standard errors relative to the main genetic parameters calculated by entry (three years of multiplication). Number of successfully analysed genotypes (N), observed (Na), and effective alleles (Ne), observed heterozygosity (Ho), expected heterozygosity (He), and Fixation Index (F) worked out for original LR, Syn1-PG, its derived populations by three years of multiplication in Central Italy and hybrid controls on the basis of data recorded overall for the 22 markers used. [file 12863_2020_846_MOESM7_ESM.pdf]

**Entry**		***N***	***Na***	***Ne***	***Ho***	***He***	***F***
LR	Mean	30.91c	3.50a	2.37a	0.48a	0.51a	0.04abc
	SE	0.262	0.327	0.212	0.044	0.039	0.054
Syn1-PG	Mean	31.32bc	3.50a	2.38a	0.46ab	0.52a	0.09abc
	SE	0.179	0.307	0.188	0.041	0.037	0.055
Syn2-PG	Mean	31.36abc	2.95ab	2.18ab	0.53a	0.49a	-0.05abc
	SE	0.124	0.223	0.154	0.045	0.034	0.068
Syn3-PG	Mean	31.91ab	3.18ab	1.87bc	0.36abc	0.43a	0.17ab
	SE	0.063	0.252	0.101	0.031	0.031	0.063
Syn2-GR	Mean	31.86ab	2.68b	1.93ab	0.39abc	0.43a	0.05abc
	SE	0.100	0.202	0.143	0.037	0.037	0.052
Syn3-GR	Mean	31.00c	2.64b	1.80bcd	0.41abc	0.38ab	-0.02abc
	SE	0.174	0.203	0.129	0.051	0.043	0.067
Syn2-TER	Mean	31.77ab	2.45bc	1.86bc	0.43abc	0.42ab	-0.03abc
	SE	0.113	0.205	0.088	0.048	0.041	0.052
Syn3-TER	Mean	31.27bc	2.73b	1.76bcd	0.31abc	0.37abc	0.24a
	SE	0.265	0.248	0.116	0.058	0.047	0.101
H	Mean	32.00a	1.50d	1.34de	0.22bc	0.18d	-0.17bcd
	SE	0.000	0.127	0.094	0.068	0.048	0.113
HH	Mean	32.00a	1.41d	1.41cde	0.41abc	0.20cd	-1.00e
	SE	0.000	0.107	0.107	0.107	0.054	0.000
HHH	Mean	32.00a	1.27d	1.19e	0.19c	0.10d	-0.51de
	SE	0.000	0.097	0.083565	0.084	0.042	0.177
HHHH	Mean	31.91ab	1.82cd	1.44cde	0.34abc	0.24bcd	-0.21cd
	SE	0.063	0.156	0.090	0.077	0.047	0.127
