# Supplementary material for: A method for obtaining flexible broccoli varieties for sustainable agriculture
Source: BMC Genet. 2020 May 7;21:51. doi: 10.1186/s12863-020-00846-2 (PMC7203864; doi:10.1186/s12863-020-00846-2)

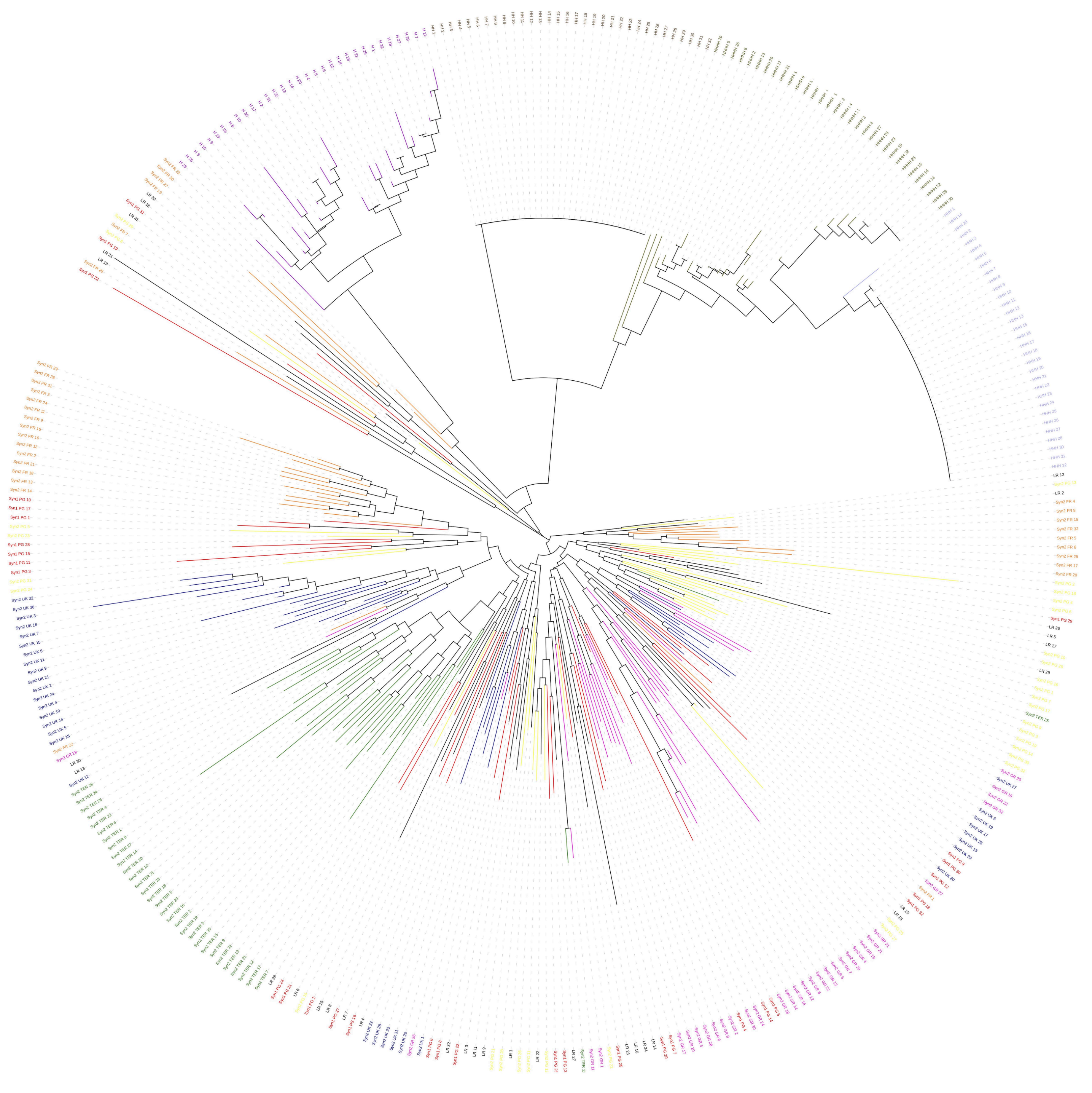

Supplement: Supplementary file 4 — Additional file 4: Figure S3. Neighbour-joining tree of genetic distances of the entries (two years of multiplication). B. oleracea var. italica original LR, Syn1-PG, its derived population by two years of multiplication across Europe and hybrid controls are coded with abbreviations and coloured with different colours as follows: LR-black, Syn1-PG-red, Syn2-PG-yellow, Syn2-GR-purple, Syn2-TER-dark green, Syn2-UK-blue, Syn2-FR-orange, H-violet, HH-brown, HHH-pink, HHHH-saddlebrown. Each individual of each entry are code whit a different number. [file 12863_2020_846_MOESM4_ESM.pdf]
